# Clinical applications of functional MRI in epilepsy

**DOI:** 10.4103/0971-3026.41829

**Published:** 2008-08

**Authors:** Chandrasekharan Kesavadas, Bejoy Thomas

**Affiliations:** Department of Imaging Sciences and Interventional Radiology, Sree Chitra Tirunal Institute for Medical Sciences and Technology, Trivandrum - 695 011, India

**Keywords:** Epilepsy, functional, MRI

## Abstract

The role of functional MRI (fMRI) in the presurgical evaluation of patients with intractable epilepsy is being increasingly recognized. Real-time fMRI is an easily performable diagnostic technique in the clinical setting. It has become a noninvasive alternative to intraoperative cortical stimulation and the Wada test for eloquent cortex mapping and language lateralization, respectively. Its role in predicting postsurgical memory outcome and in localizing the ictal activity is being recognized. This review article describes the biophysical basis of blood-oxygen-level-dependent (BOLD) fMRI and the methodology adopted, including the design, paradigms, the fMRI setup, and data analysis. Illustrative cases have been discussed, wherein the fMRI results influenced the seizure team's decisions with regard to diagnosis and therapy. Finally, the special issues involved in fMRI of epilepsy patients and the various challenges of clinical fMRI are detailed.

Functional MRI is a technique that maps the physiological or metabolic consequences of altered electrical activity in the brain. In contrast to positron emission tomography (PET), a similar brain mapping technique and one that has been used for many years to study brain function, fMRI is not based on ionizing radiation and thus can be repeated as often as is necessary in patients or normal volunteers. Electroencephalography (EEG) and magnetoencephalography (MEG) map the electrical activity in the brain. Although EEG and MEG have high temporal resolution (10-100 milliseconds), they suffer from poor spatial resolution (one to several centimeters). The blood-oxygen-level-dependent (BOLD) fMRI technique has a spatial resolution of a few millimeters and a temporal resolution of a few seconds.[1]

## Biophysical Basis of BOLD fMRI

Neuronal stimulation leads to a local increase in energy and oxygen consumption in functional areas. The subsequent local hemodynamic changes transmitted via neurovascular coupling are measured by fMRI. The close coupling between regional changes in brain metabolism and regional cerebral blood flow (CBF), called ‘activation flow coupling’ (AFC), was originally described by Roy and Sherrington in 1890.[2] The BOLD technique depends on the difference in the magnetic properties between oxygenated (oxy-Hb) and deoxygenated (deoxy-Hb) hemoglobin. The ferrous iron on the heme moiety of deoxy-Hb was shown to be paramagnetic by Thulborn and colleagues in 1982.[3] Paramagnetic deoxy-Hb produces local field inhomogeneities in the measurable range of MRI, resulting in signal decrease in susceptibility-weighted MRI-sequences (T2*), whereas diamagnetic oxy-Hb does not interfere with the external magnetic field. Ogawa and coworkers working on a rat model at 7 Tesla showed that the oxygenation of blood has a measurable effect on the MRI signal.[4] Kwong *et al*, in 1992, demonstrated that brain activation in human subjects produced a local signal increase that could be used for functional brain imaging.[5] In the same year, several others reported similar findings.[6–8]

**Figure 1:**
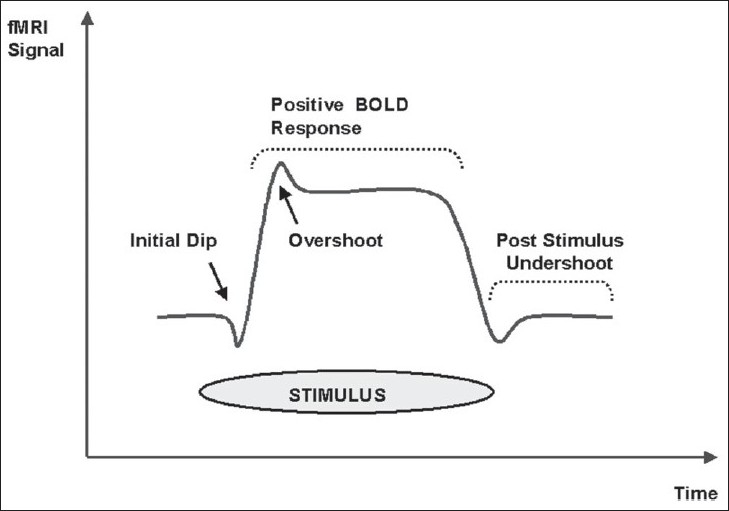
BOLD signal shows initial dip and then a more prolonged ‘positive’ signal

When the neurons are stimulated there is an increase in local oxygen consumption that results in an initial decrease of oxy-Hb and an increase in deoxy-Hb in the functional area. To provide the active neurons with oxygenated blood, perfusion in capillaries and draining veins is enhanced within several seconds. As a result of this process, the initial decrease of local oxy-Hb is equalized and then overcompensated.[9] The deoxy-Hb is progressively washed out. This causes a reduction of local field inhomogeneity and an increase of the BOLD signal in T2*W MRI images[10] [Figure 1]. Although the ‘initial dip’ corresponds to the neuronal activity both temporally and spatially, this is more difficult to measure in clinical settings.[11] Electrophysiologically, it is the local field potential that changes with an increase in the BOLD signal and not the neuronal firing rate.[12] 

## Design of fMRI Experiments and Data Acquisition

**Figure 2:**
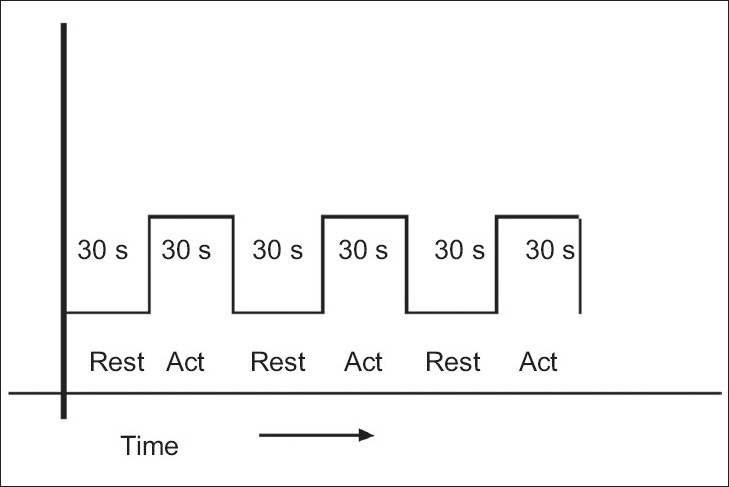
Boxcar design: Rest and active conditions are repeated alternatively during the acquisition

The most common imaging sequence used in fMRI studies is echoplanar imaging (EPI).[13] This is a very fast MRI imaging sequence, which can collect whole brain data within a few seconds. However, the spatial resolution is significantly lower than in anatomic MRI images. Also EPI images are sensitive to field inhomogeneities, leading to geometric distortion of the images in certain brain regions. In a typical fMRI experiment, a large set of images is acquired very quickly, while the patient or subject performs a task that shifts brain activity between two or more well-defined states (boxcar design) [Figure 2].

The signal time course in each voxel of the slices and the time course of different tasks are correlated. This can identify voxels in brain that show statistically significant changes associated with the brain function under consideration.[1] Later these statistical maps (Z scores) are superimposed on a high-resolution anatomic image by using a coregistration technique for proper identification of the precise anatomic location of the origin of the signal. Although this appears complicated, most of this can now be done online using the real-time fMRI packages available in newer MRI machines.

Most of the clinical fMRI experiments use a boxcar or block design. It is the simplest and the most time-efficient approach for comparing brain response in different states. In this design, for relatively long periods (e.g., 30 s), a discrete cognitive or motor state is maintained (in the simplest form, two states: rest *vs* activity) and is alternated during scanning. Since this is not a physiological design (i.e., it is an artificial state), some tasks may not be suitable for this design.[1] 

**Figure 3:**
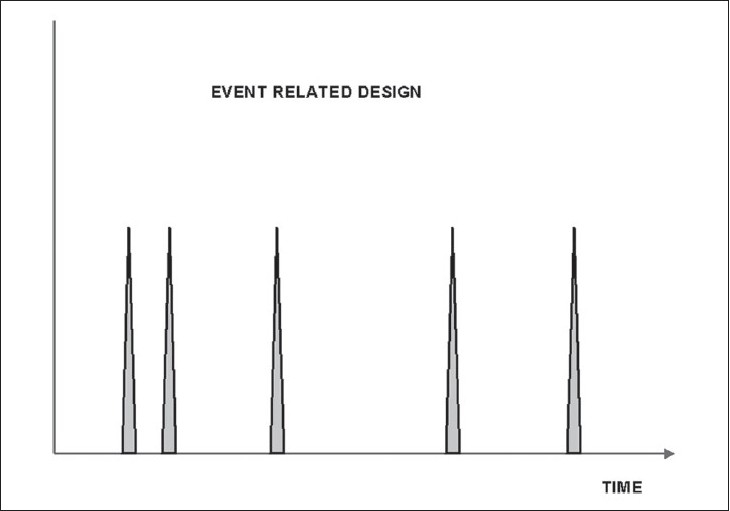
Event-related design: Stimuli repeated at variable intervals

An alternative approach, which is more physiological, is an ‘event’-related paradigm [Figure 3], in which discrete stimuli are repeated at variable times while scanning is in progress. However, this design needs longer acquisition times and is statistically more difficult to analyze and, hence, is used less often in clinical practice.

## fMRI Setup

**Figure 4:**
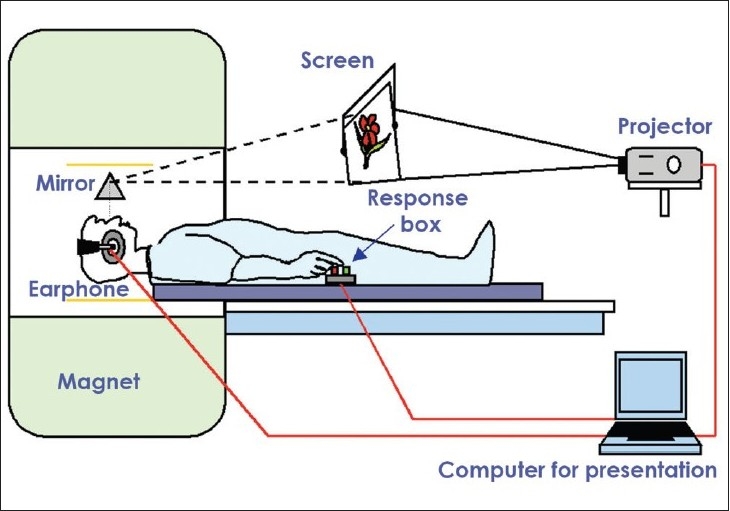
fMRI setup: Patient responds to the visual/auditory stimuli using the response box

fMRI applications in research laboratories can have permanent test setups. Here the results need not be immediately available. In contrast, fMRI in clinical / hospital settings, needs custom-tailored hardware, software, imaging protocols, and data evaluation techniques. A real-time fMRI processing tool is useful so that the results are available immediately. In a clinical setting we have to examine patients with existing deficits, and subjects may include uncooperative or sedated patients and children. At our institute we have set up a patient-friendly audiovisual projection system with a response box and synchronization device (synchronizes the visual/auditory stimulation with the MRI pulse). Figure 4 illustrates the setup that we use for clinical studies.

## fMRI Paradigms

By using optimized and standardized protocols, fMRI examinations can be integrated into routine MRI imaging without major problems. To investigate motor function, self-triggered movements are most commonly used. Motor cortex mapping is done using paradigms that include tongue movements as well as finger and toe movements, contralateral to the side of the lesion, to localize the motor homunculus in relation to the lesion. Bilateral finger movement can help in comparing the ipsilateral motor cortex with that on the opposite side. To keep the likelihood of motion artifacts to a minimum,[14,15] the following movement tasks are chosen: repetitive tongue movements, with closed mouth; opposition of fingers to thumb, with free choice of sequence; and repetitive flexion and extension of all five toes, without moving the ankle. Alternatively, in cases of mild paresis of the upper extremity, fist clenching/ releasing can be tested. The somatosensory functional areas can be studied by nonstandardized tactile stimuli (e.g., manual stroking of the hand by the examiner).[16]

Language functions are examined using various paradigms involving auditory or visual stimulation. A task commonly and easily performed in patients for the purpose of lateralization is the “verb generation task” (also called “verbal fluency task”). This task shows relatively consistent activation of the anterior language areas. Another task, the “semantic decision-making task”, demonstrates more widely distributed networks, including the anterior and posterior language areas.[17] The "visual stimulation task" is performed by showing checkerboards during the active period and a blank screen during the rest period.

## Data Analysis

**Figure 5:**
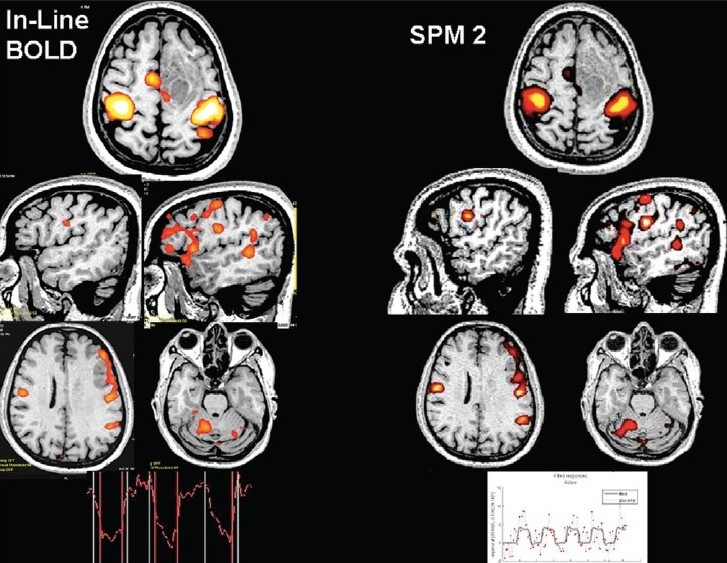
Real-time fMRI (vendor provided - Inline BOLD) *vs* offline processing using statistical parametric mapping (SPM). Inline BOLD fMRI coregistered on 3D-FLASH images with a time-activity curve of a patient with seizures due to a frontal mass lesion, obtained after bilateral finger tapping and verb generation task, is compared to the fMRI results with a time-activity curve after offline processing using SPM2 of the same data set and subsequent coregistration onto 3D-FLASH images using MRIcro software. Note the similarity in activation with real-time processing using inline BOLD and offline processing techniques using SPM2

Each fMRI experiment generates a huge amount of data, which needs to be analyzed rigorously in order to obtain the best results. As mentioned earlier, for simple analysis, real-time fMRI processing will help. Our earlier studies have shown that real-time fMRI analysis by vendor-provided fMRI processing tool can give clinically useful information comparable to the time-tested postprocessing tools[18] [Figure 5]. Presently, we perform most of our fMRI studies using real-time fMRI processing. The fMRI results are then coregistered on 3D-FLAIR images. We have found coregistering on 3D-FLAIR more useful than on T1W 3D spoiled gradient images (3D FLASH/3D SPGR).[18] 

For event-related paradigms and more complex boxcar paradigms involving more than two states, extensive computation may be required using any of the free or commercial softwares, such as statistical parametric mapping (SPM) (www.fil.ion.ucl.ac.uk/spm), FSL ( www.fmrib.ox.ac.uk/fsl) or Brain Voyager ( www.brainvoyager.de).The basic idea of analysis of functional imaging data is to identify voxels that show signal changes that vary with the changes in the given cognitive or motor state of interest, across the time course of the experiment. This is quite a challenging problem as the fMRI signal changes are very small (of the order of 0.5-5%), leading to a high probability of false negative results. The chance of false positive activation is also very high. Different types of analysis like ‘fixed effects,’ ‘random effects,’ or ‘mixed effects’ can be undertaken.[1] 

The greatest problem during any fMRI experiment is subject motion. The BOLD signal is extremely sensitive to motion, which can spoil the whole experiment. Motion can be gross head movement or even the minimal brain motion associated with cardiac or respiratory cycles. Most of the analysis software includes some realignment and coregistration programs to minimize motion effects. Another step is performed using spatial smoothing and temporal filtering, to reduce the noise in the data. The images can then be normalized to a common brain space [e.g., Montreal Neurological Institute (MNI) template]. This step is utilized mostly in research studies and is not needed for individual patients in the clinical setting. Various statistical tests can then be applied on a voxel-by-voxel basis to test the significance of a particular voxel with an increased signal associated with a certain brain state. The commonly used method is ‘t’ statistics. The ‘t’ statistical maps are then superimposed on high-resolution anatomical images to obtain a clinically useful fMRI output.

## Clinical Applications in Patients with Epilepsy

fMRI has been used to study patients with a broad range of neurological disorders and across a wide spectrum of disease severity. The results have provided insights into the mechanism of disease as well as into normal brain function. The majority of the studies published have been performed in research settings. The clinical role of fMRI is being increasingly recognized. One of the earliest and best-validated clinical applications of fMRI was, and remains, presurgical assessment of brain function in patients with brain tumors and epilepsies. There is a substantial body of evidence that shows that fMRI is a good technique for localizing different body representations in the primary motor and somatosensory cortex, as well as for localizing and lateralizing language function prior to surgery. This diagnostic information permits function-preserving and safe treatment. We illustrate the clinical applications through patients whom we have investigated at our hospital during the last three years.

## Mapping the Eloquent Cortex

Mapping eloquent areas can be done using invasive methods such as intraoperative cortical stimulation in awake patients, implantation of a subdural grid, or intraoperative recording of sensory-evoked potentials.[19,20] fMRI can obtain these data preoperatively and noninvasively. Together with its high sensitivity for visualizing brain lesions, fMRI can define the relation between the margin of a lesion and any adjacent functionally significant brain tissue. fMRI has the potential to predict possible deficits in motor and sensory perceptual functions or in language that would arise from intrinsic lesion expansion or from therapeutic interventions such as surgery. This helps in decision making during patient management. The relative risk of intervention *vs* nonintervention can be discussed and explained to the patient and the relatives. Further, a decision on the treatment option to be adopted can be made after considering the cost of treatment and the benefits that can be expected from the treatment.

Earlier studies aimed at comparing intraoperative corticography (ECoG) with fMRI revealed good spatial correlation between the two methods.[21–23] A few studies have also tried to assess the chances of postoperative deficits when a lesion was placed in, or was close to, the eloquent cortex.[24] Lee *et al*, evaluated the ways in which fMRI studies have influenced patient management. In patients with medically refractory epilepsy, fMRI results helped to assess the feasibility of resection in 70%, to plan the surgical procedure in 43%, and to select patients for invasive mapping in 52%.[25]

**Figure 6:**
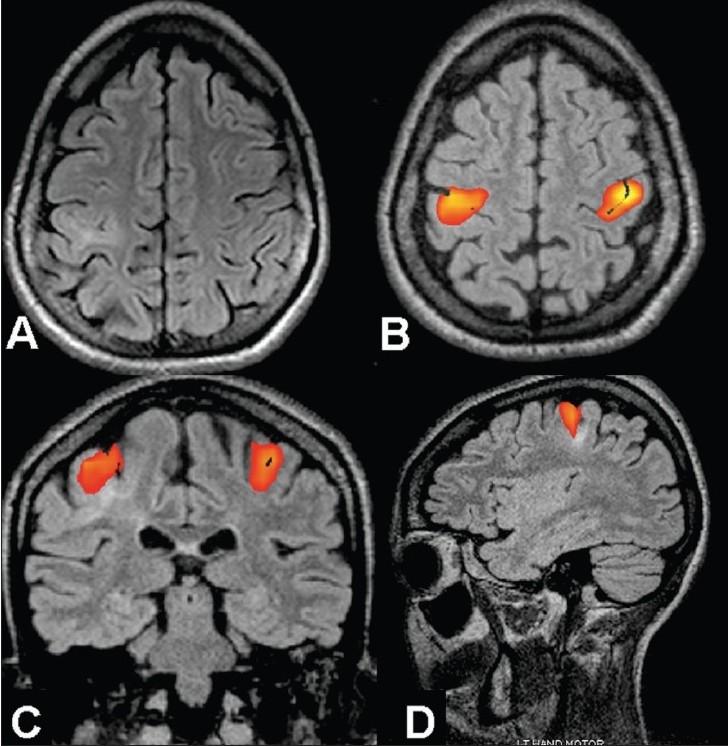
An 8-year-old female presented with left focal motor seizures since the age of 4 years. The frequency of seizure had increased over the last six months to the current frequency of about 4-5 seizures per month. Axial FLAIR MRI image (A) reveals a thickened cortex with a widened sulcus and underlying white matter hyperintensity, suggestive of focal cortical dysplasia in the right sensorimotor cortex. Inline BOLD fMRI coregistered on 3D-FLAIR axial (B), coronal (C), and sagittal (D) images obtained after bilateral finger tapping *vs* rest show that the primary motor hand area is lying within the lesion. The likelihood of left limb weakness after surgery was explained to the parents. Since the seizures could be controlled better with the addition of newer drugs, it was decided to keep the patient on follow-up

**Figure 7:**
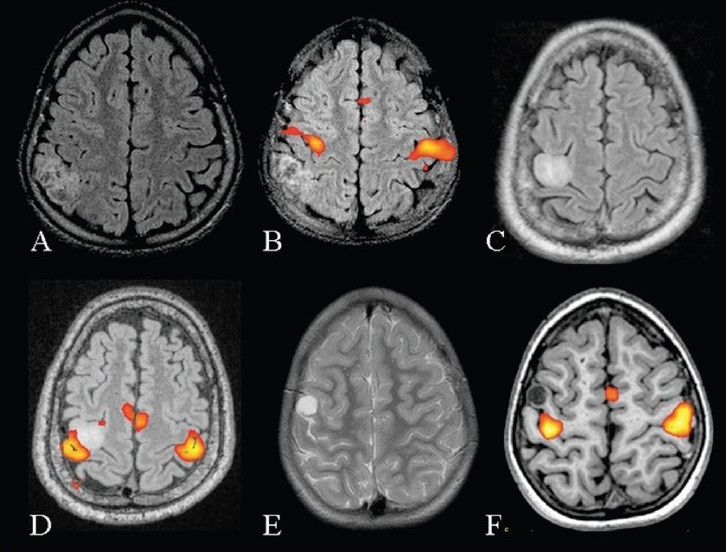
Three patients with intractable seizures due to a mass lesion close to the sensorimotor cortex. Presurgical mapping of the motor cortex was performed using bilateral finger tapping *vs* rest, in all the three patients. MRI (axial FLAIR) and fMRI (inline BOLD coregistered on FLAIR) images (A, B) of the first patient show that the lesion is posterior to the right postcentral gyrus. Since the lesion was placed well away from the motor cortex it was decided to proceed with surgery. MRI and fMRI in the second patient (C, D) show the mass lesion to be abutting the right hand area. The postsurgical risk of developing limb weakness was explained to the patient. MRI and fMRI images of the third patient (E, F) show that the lesion is placed in the motor cortex lateral to the right hand area. Fortunately the seizures in this patient could be controlled with antiepileptic medication. It was decided to postpone the surgery and keep the patient on regular follow-up

**Figure 8:**
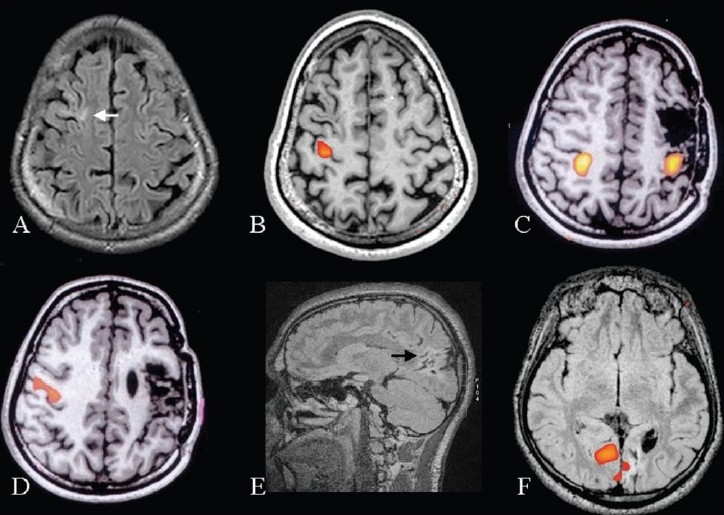
Three patients with seizures due to a gliotic area. In the first patient, the gliosis was due to an old healed granuloma. Axial FLAIR and inline BOLD coregistered on 3D-FLASH (A, B) images, obtained after left hand movement *vs* rest, show that the right motor hand area is well away from the gliotic area (white arrow). In the second patient, the gliosis was postsurgical. Inline BOLD fMRI coregistered on 3D-FLASH images, obtained after bilateral hand movement *vs* rest and tongue movement *vs* rest (C, D) show that the left hand area is placed closer to the gliotic area, while the face area on the left side is not seen. The left hand activation area is pulled towards the gliotic area. The third patient showed gliosis in the occipital cortex probably secondary to perinatal hypoglycemia. Sagittal FLAIR and inline BOLD fMRI coregistered on axial FLAIR images obtained after visual stimulation show minimal activation in the gliotic left occipital cortex. Most of the visual activation is from the right side. All the three patients, in whom fMRI helped in surgical planning, underwent resection of the gliotic area without developing neurological deficits

In our series of patients, fMRI results matched those from intraoperative cortical stimulation, for lesions in, or close to the eloquent cortex. They also matched Wada test results for language hemispheric dominance.[18] Eloquent cortex mapping was performed in epilepsy patients with tumor, gliosis, or malformation of cortical development in, or close to the eloquent cortex. Our neurosurgeons have found fMRI for eloquent cortex mapping most useful in patients with gliosis, in whom the distortion in anatomy makes prediction of the eloquent cortex extremely difficult. Usually gliotic lesions pull the functionally active areas towards them. Space-occupying lesions such as tumors of the brain primarily displace the functional cortex. For this reason, resection within the boundaries of a lesion should not directly damage the eloquent cortex and result in a significant deficit. In contrast, functional reorganization may or may not happen within the dysplastic cortex in malformations of cortical development. Our study using fMRI on cortical malformations showed that functional reorganization is unpredictable in these lesions.[26] The dysplastic cortex can retain useful brain function. The following three cases illustrate the usefulness of fMRI in selecting patients for surgery, tailoring surgical resection, and in predicting the postsurgical outcome [Figures 6–8].

## Lateralization of Language Functions

Intracarotid amobarbital testing (Wada testing) has been the gold standard for identifying lateralization of language and memory functions preoperatively, but it is invasive and therefore carries a small, but definite risk of complications. fMRI offers a promising noninvasive alternative approach.[27] While there is good agreement between the Wada and fMRI results, fMRI is more sensitive to involvement of the nondominant hemisphere. Binder *et al*,[28] reported a cross-validation study comparing language dominance determined by both fMRI and the Wada test in 22 patients. The majority of studies opine that semantic decision tasks should be used rather than verbal fluency tasks because the latter may lack the ability to activate the posterior language areas.

**Figure 9:**
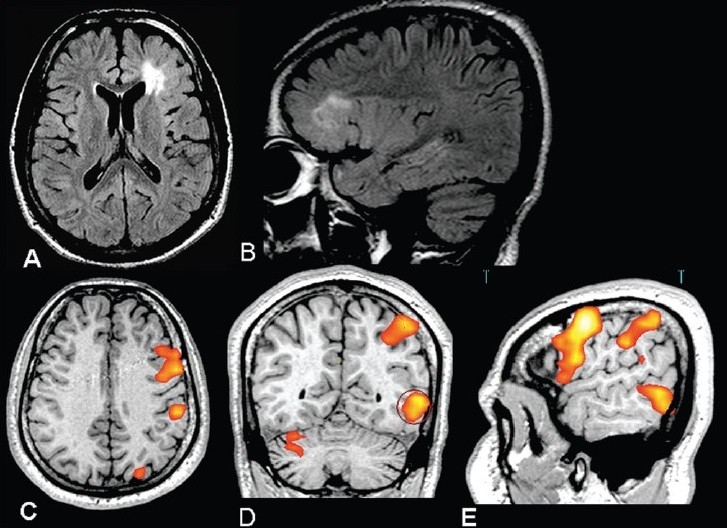
This 22-year-old right-handed male was referred for surgical treatment of medically refractory partial seizures that he had been suffering from the age of 3 years. The video-scalp EEG monitoring confirmed that the origin of the seizures was from the left frontal lobe. Axial (A) and sagittal (B) FLAIR images show a thickened cortex, poor grey-white distinction and underlying white matter hyperintensity in the left frontal area suggestive of focal cortical dysplasia. Inline BOLD fMRI, language area mapping using verb generation task coregistered on axial (C), coronal (D), and sagittal (E) 3D-FLASH images, shows strong left lateralization of language. The lesion is adjacent to the Broca's area. The fMRI helped to define Broca's area, which was preserved during a tailored surgical resection with no postoperative expressive speech deficit. Histopathology confirmed cortical dysplasia

**Figure 10:**
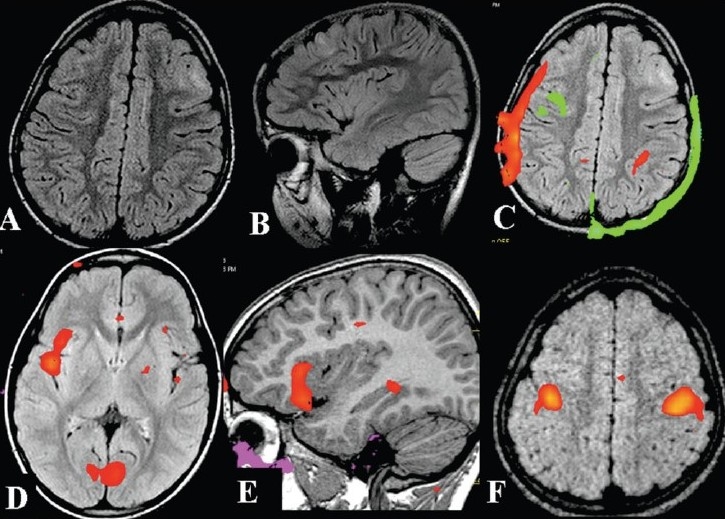
A 6-year-old boy with a history of unprovoked seizures from one-and-a-half years of age was put on different antiepileptic drugs without any significant benefit. Video-electroencephalogarphy showed eight complex partial seizures of left frontal origin. The axial (A), and sagittal (B) FLAIR MRI brain images reveal a thickened cortex with hyperintensity in the left frontal area underneath the coronal suture suggestive of focal cortical dysplasia. Before lesionectomy, there was a need for an fMRI study to map the distance of the lesion from the motor cortex and the language area. Inline BOLD fMRI performed using a verb generation task before training (C) and after training (D, E) shows that the language is lateralized to the right side. No interpretation is possible in the fMRI performed before training because of the patient's head movement. On bilateral finger tapping *vs* rest the motor hand area is seen to be well away from the lesion (F). The boy underwent complete lesionectomy about 8 months back. As expected from the fMRI results, the child did not develop any postoperative neurological deficits and presently there is good seizure control

We have noted that visual presentation of the language paradigm gives much better and consistent results as compared to auditory presentation. Secondly, in a multilingual country like India, auditory language tasks may have to be modified according to the primary language of the patient. This can be solved to some extent by showing the nouns as pictures in the verb generation task. In our fMRI language studies we perform both the semantic decision task and the verbal fluency task by visual presentation. The former is done using a discrimination task of word pairs - related/unrelated, judging the meaning of sentences, and identifying grammatically accepted language. These tasks are preferably done in the primary language of the patient. The following two cases illustrate the usefulness of fMRI in language lateralization [Figures 9 and 10].

## Memory

Performing fMRI to map memory is more challenging than mapping language. fMRI has been found to be useful in predicting postoperative memory deficits. Memory processing involves encoding and retrieval of face, patterns, words, sceneries, etc. Paradigms for each of these tasks show activation in different areas. It is also difficult to separate brain activity related to memory from that related to other cognitive processes.[29] Detre *et al*, were the first to demonstrate that fMRI could be used to detect clinically relevant asymmetries in memory activation in patients with temporal lobe epilepsy.[30] In a study by Golby *et al*, fMRI was used to study the lateralization of memory encoding processes (patterns, faces, scenes, and words) within the mesial temporal lobe in patients with temporal lobe epilepsy.[31] Rabin *et al*, used a complex visual scene-encoding task that causes symmetrical mesial-temporal-lobe activation in controls, to determine a relationship between mesial temporal lobe activation asymmetry ratios and postsurgical memory outcome.[32] It was shown that increased activation ipsilateral to the seizure focus is associated with greater memory decline. A more recent study has shown similar results.[33] We have developed simple memory encoding paradigms that can be used in Indian patients with epilepsy. We have tested these in controls and have found the results to be consistent.

## Localizing Spontaneous Ictal Activity

Using a newer technique that allows concurrent EEG and fMRI, it is possible to localize the regional metabolic changes accompanying ictal activity.[34,35] These techniques capitalize on the temporal resolution of EEG and spatial resolution of fMRI. The approach of concurrent EEG and fMRI recording tends to be more efficient and accurate as compared to the spike-triggered approach. These techniques may be of particular value in presurgical evaluation of neocortical epilepsy, where paroxysmal activity on EEG may remain poorly localized. In addition, these techniques may provide new insights into the anatomical and pathophysiological correlates of unifocal and multifocal spike discharges.

The MRI scanner is a hostile environment for EEG recordings. MR-compatible EEG recording equipment must ensure patient safety, sufficient quality of the EEG signals, and avoid compromising MRI image quality. Technical issues related to EEG-correlated fMRI have been addressed in detail in several previous articles[36] EEG-correlated fMRI has been shown to be a practicable method in epilepsy patients with frequent interictal epileptiform discharges on scalp EEG.[37,38] A recently published study has evaluated the clinical usefulness of this technique in presurgical localization of the epileptogenic focus.[39] 

## Challenges for Presurgical fMRI

1. Patients with epilepsy on long-term antiepileptic medication and those who have frequent seizures can have low intelligence quotients (IQ). These patients may not co-operate for difficult tasks such as the language and memory tasks. They may, however, be able to perform simpler motor tasks. Before fMRI is performed, each of our epilepsy patients undergoes a neuropsychology test for assessing the IQ.[18]
2. The effects of medication on the BOLD signal response have not been systematically studied as yet. In a study by Jokeit *et al*,[40] the extent of fMRI activation of the mesial temporal lobes induced by a task based on the retrieval of individual visuospatial knowledge was correlated with the serum carbamazepine level in 21 patients with refractory temporal lobe epilepsy. The study showed that the carbamazepine level can significantly influence the amount of fMRI activation.
3. Ictal and interictal epileptic activity in a patient with epilepsy can influence the lateralization of mesiotemporal memory functions and language functions.[41,42] The next three challenges mentioned are some of the general challenges for clinical fMRI.[43]
4. Head motion: Signal intensity changes observed in fMRI images are small. These may be contaminated by gross head motion. Additional minor contamination results from physiologic brain motion (pulsation of the brain, overlying vessels, and cerebrospinal fluid). Head movement during the acquisition phase can be restricted by fixation of the head with straps. However we have found that patients find this uncomfortable. Postprocessing techniques in the offline tools, like realignment and coregistration can help in correcting for head movement. Stimulation paradigms that induce less patient head motion are preferred. Finally, patient cooperation is an essential element both in task compliance and in restricting head motion. If we are planning to do a routine MRI brain study along with fMRI, it is better to do the fMRI study first when patient cooperation is better. In patients in whom we have performed fMRI immediately after the routine brain study we have seen the head movement to be more. Secondly, adequate training before imaging could increase the familiarity with the imaging process. We have found training to be extremely useful in pediatric fMRI and we have been able to do fMRI studies for language lateralization in children as young as 5-6 years of age [Figure 10].
5. There is a concern that fMRI examinations at a field strength of 1.5 Tesla images predominantly large, draining veins. Gao *et al*, have shown that fMRI images weighted toward the microcirculation may be obtained at 1.5T, if the pulse sequence is designed for minimizing inflow effects and maximizing BOLD contribution.[44] Maximizing the fMRI signal toward the site of neuronal activity can also be achieved by optimizing the mode of stimulation as shown by the study of Le Rumeur *et al.*[45]
6. Does the absence of a BOLD signal in a cortical area indicate with certainty a lack of electrical neuronal activity in that area? Different pathologic conditions could weaken the hemodynamic response that is the source of the fMRI signal. Examples of this include peritumoral vasogenic edema producing mechanical vascular compression and drugs administered to the patient causing change in the hemodynamic autoregulation.

## Conclusions

Mapping sensorimotor, visual, language, and memory function using fMRI can identify the eloquent cortex and predict postoperative deficits of specific functions during the presurgical workup of patients with epilepsy. In selected patients with frequent interictal epileptiform discharges, EEG-correlated fMRI has the potential to identify the cortical areas involved in generating the discharges. Better and better techniques are slowly evolving to solve challenges in clinical fMRI. With the availability of higher Tesla magnets, faster sequences, and better paradigms and postprocessing tools, the clinical application of this technique in patients with epilepsy is going to increase in the years to come.
